# Overview of NIA Research and Priorities

**DOI:** 10.1093/geroni/igab046.1216

**Published:** 2021-12-17

**Authors:** Richard Hodes

**Affiliations:** National Institute on Aging, Bethesda, Maryland, United States

## Abstract

Dr. Hodes will provide an overview of NIA's structure and mission, in addition to discussing research foci from across the Institute's scientific divisions.

